# Conjunctival Advancement Procedure for Repairing Conjunctival Exposure of the PreserFlo MicroShunt

**DOI:** 10.7759/cureus.72409

**Published:** 2024-10-26

**Authors:** Masaki Tanito, Mizuki Iida, Kana Murakami, Chisako Ida, Hinako Otani, Keigo Takagi, Akiko Harano, Sho Ichioka, Kazunobu Sugihara

**Affiliations:** 1 Department of Ophthalmology, Shimane University Faculty of Medicine, Izumo, JPN

**Keywords:** conjunctival advancement procedure, device erosion, glaucoma surgery, preserflo™ microshunt, surgical repair

## Abstract

A 59-year-old Japanese woman with primary open-angle glaucoma underwent PreserFlo MicroShunt (PFM) (Santen Pharmaceutical Co., Ltd., Osaka, Japan) implantation in her left eye. Six weeks postoperatively, a conjunctival exposure of the posterior end of the device was observed, accompanied by aqueous leakage. The patient had no identifiable risk factors for exposure, such as previous conjunctival incisional surgeries, significant blepharitis, or needling procedures. Conjunctival advancement surgery was performed to cover the exposed device. After the sufficient dissection of scar tissue surrounding the PFM and the reapproximation of the conjunctiva, the device was successfully covered. Postoperatively, the filtration bleb remained formed, and the intraocular pressure (IOP) was controlled. No recurrence of device exposure was observed after 18 months of follow-up. Although PFM device exposure is a rare complication, it requires prompt surgical intervention to prevent infection and maintain IOP control. Conjunctival advancement surgery is an effective treatment option that allows for the repair of the exposure while preserving the filtration function of the device.

## Introduction

The PreserFlo MicroShunt (PFM) (Santen Pharmaceutical Co., Ltd., Osaka, Japan) is a glaucoma surgical device used in filtration surgery and designed to reduce intraocular pressure (IOP) by draining aqueous humor from the anterior chamber to the subconjunctival space [1,2]. While PFM may be less effective in lowering IOP compared to trabeculectomy [3], it offers the advantage of a simpler surgical procedure, as there is no need to create a scleral flap or perform an iridectomy [1,2]. Recent studies have reported a similar safety profile between PFM and trabeculectomy, with no significant difference in IOP reduction [4,5]. Furthermore, the re-intervention rate following filtration surgeries is lower with PFM compared to the Xen Gel Stent [6].

Device exposure following PFM implantation is a relatively rare complication, although several cases have been documented [7-9]. If left untreated, device exposure poses a risk of intraocular infection, making prompt surgical intervention essential. Treatment options reported include device removal [8,9], relocation to a different site [7], and coverage with an amniotic membrane graft [8]. Here, we present a case of PFM exposure through the conjunctiva, successfully treated using an anterior conjunctival advancement procedure.

## Case presentation

A 59-year-old Japanese woman was referred to Shimane University Hospital for the treatment of glaucoma in both eyes. She had previously undergone bilateral ab interno Tanito microhook trabeculotomy four years earlier in both eyes. She was on antihypertensive medications but had no other significant systemic disease. Her current ocular medications included latanoprost eye drops and a combination of timolol-dorzolamide eye drops for both eyes. Her best-corrected visual acuity (BCVA) was 1.2 in both eyes with a -4.5 D spherical lens. IOP measured by Goldmann applanation tonometry was 14 mmHg in the right eye and 20 mmHg in the left eye.

The examination of the anterior segment (AS) revealed no conjunctival scars or inflammation in either eye. The corneas were clear, and the anterior chambers were deep, without signs of inflammation. The lenses were clear. Gonioscopy showed grade 4 open angles (Shaffer classification) with mild pigment deposition. The nasal trabeculotomy sites from the previous surgery were visible, though the trabeculotomy clefts were indistinct. There was no peripheral anterior synechiae. The vertical cup-to-disc ratios were 0.8 in the right eye and 0.9 in the left eye, with a wedge-shaped nerve fiber layer defect (NFLD) observed in the left eye. Humphrey visual field testing (30-2 program, Carl Zeiss Meditec, Dublin, CA) showed a mean deviation of +2.0 dB in the right eye and +1.22 dB in the left eye, with a corresponding visual field defect consistent with the NFLD in the left eye. Central corneal thickness was 533 μm in the right eye and 512 μm in the left eye. The patient was diagnosed with ocular hypertension in the right eye and primary open-angle glaucoma in the left eye.

Surgery was performed in the superonasal quadrant with a fornix-based conjunctival incision. Intraoperatively, 0.04% mitomycin C (MMC) was applied under the conjunctiva for three minutes and irrigated with a balanced salt solution. A specialized double-step knife was used to create a scleral tunnel 3 mm posterior to the limbus, through which the PFM was inserted. Aqueous humor outflow from the PFM was confirmed using a surgical sponge. Tenon’s tissue and conjunctiva were reapproximated to the limbus with 10-0 Vicryl sutures (Johnson & Johnson, New Brunswick, NJ), completing the procedure. No conjunctival fistula was observed intraoperatively. Intraoperatively, no tearing of Tenon’s capsule or conjunctiva was observed. Postoperatively, levofloxacin and betamethasone eye drops were prescribed four times daily.

**Figure 1:**
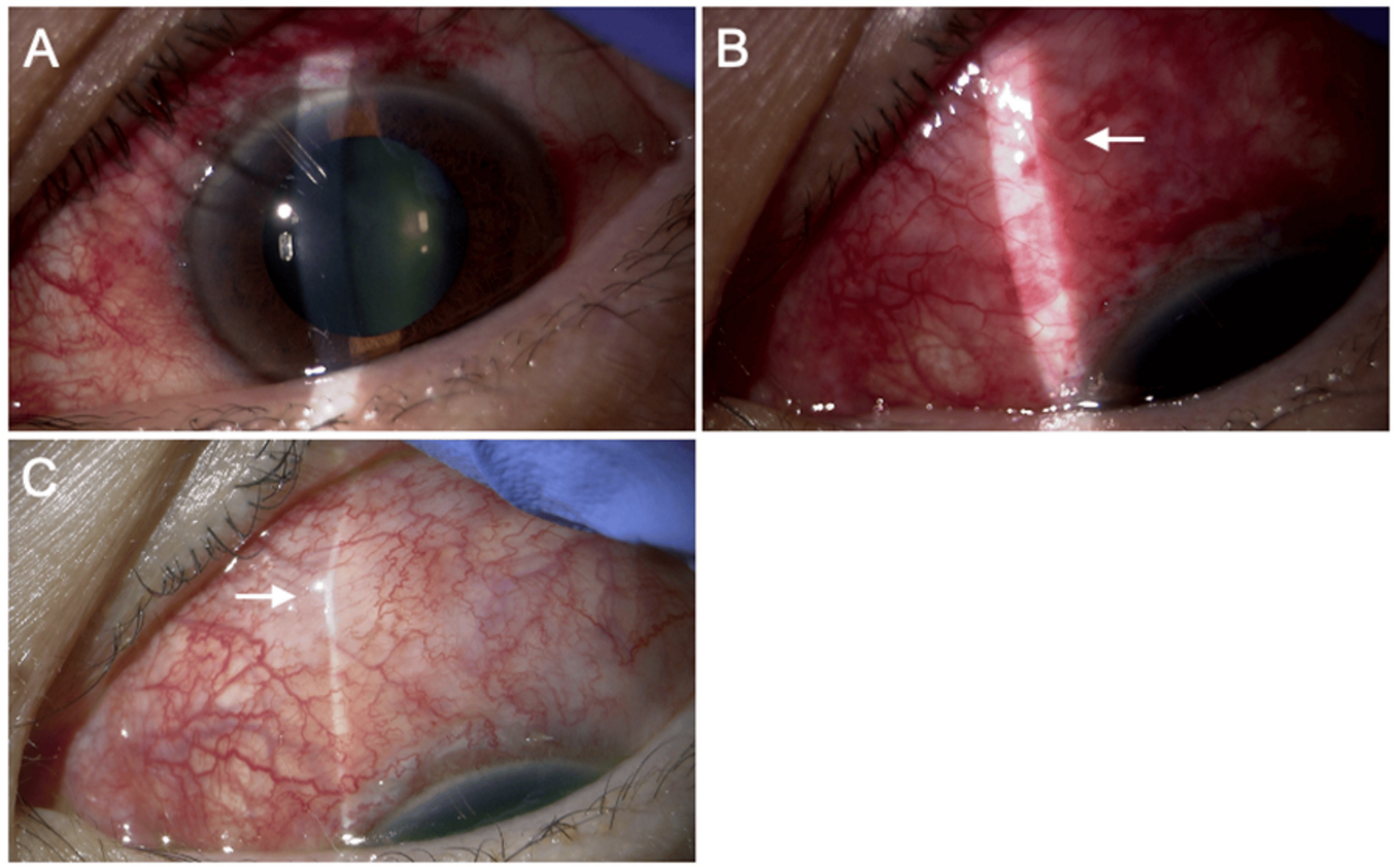
Slit lamp findings two days (A and B) and seven days (C) after the PFM implantation. (A) The tip of the PFM is seen in the anterior chamber at the superior nasal quadrant OS. (B and C) The protrusion of the conjunctiva caused by the distal end of the PFM is observed, but device exposure is not present (arrow). PFM, PreserFlo MicroShunt; OS, oculus sinister

On the first postoperative day, the anterior chamber was deep, and hyphema was minimal, scoring 001 on the Shimane University Hyphema Scoring System [10]. By the second postoperative day, bleb formation was satisfactory, and the IOP was 5 mmHg without glaucoma medication (Figure 1A, 1B). By the seventh postoperative day, the BCVA was 1.2, and the IOP in the left eye was 6 mmHg. Slit lamp examination revealed mild conjunctival protrusion corresponding to the posterior end of the PFM, but no device exposure was observed (Figure 1C).

**Figure 2:**
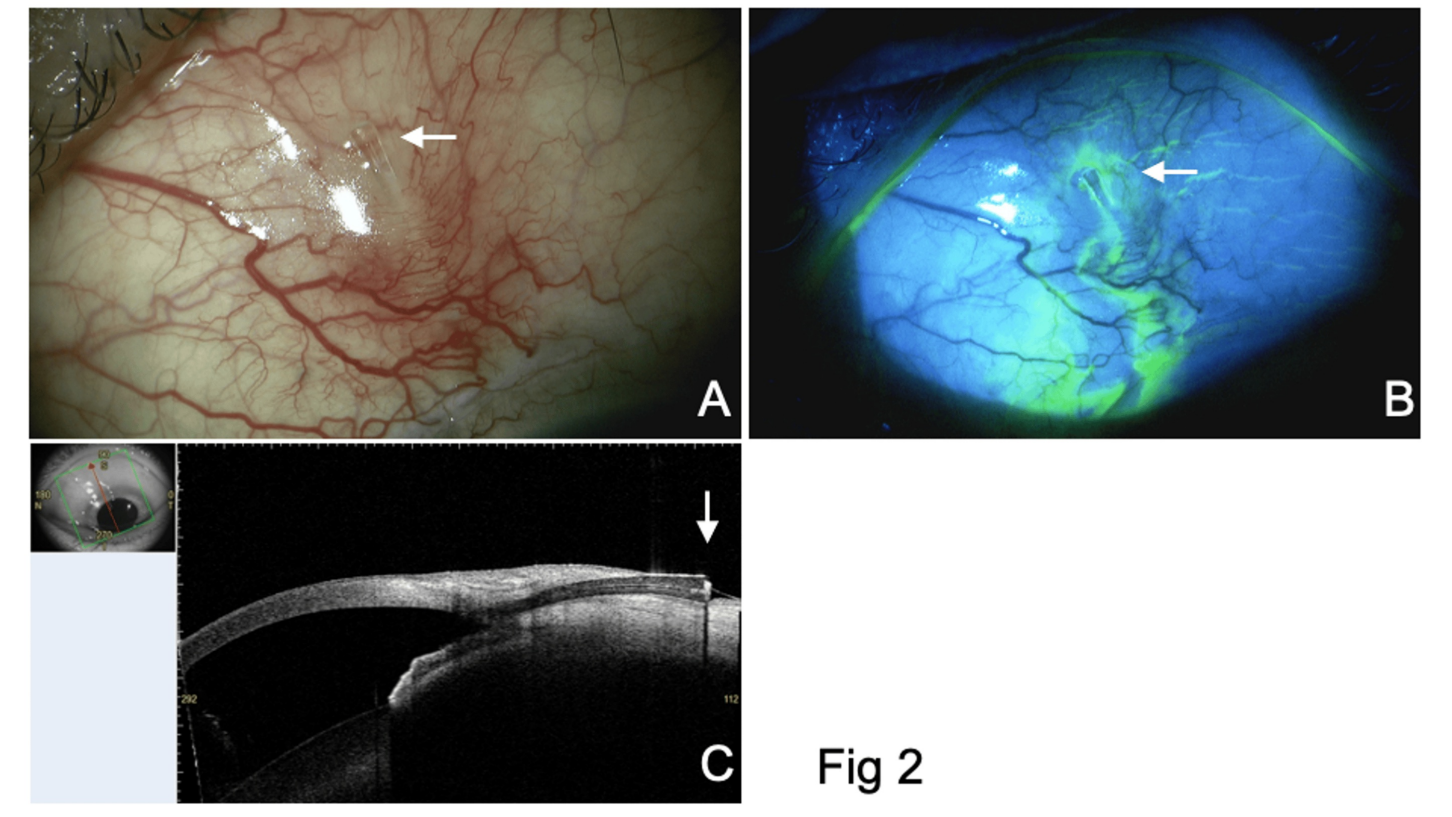
Slit lamp (A and B) and AS-OCT (C) findings six weeks after the PFM implantation. (A) The distal end of the PFM is exposed on the conjunctiva (arrow). (B) Aqueous leakage from the PFM is confirmed by fluorescein staining. (C) The distal end of the PFM is exposed on the conjunctiva (arrow). The small image in the top left of this panel shows the scan direction. PFM, PreserFlo MicroShunt; AS-OCT, anterior segment optical coherence tomography

At the six-week postoperative follow-up, the BCVA remained at 1.2, and the IOP was 9 mmHg in the left eye. Slit lamp examination revealed the exposure of the posterior end of the PFM on the conjunctiva (Figure 2A). Fluorescein staining confirmed aqueous leakage from the posterior end of the PFM to the ocular surface (Figure 2B and Video 1). The anterior chamber was deep, without signs of inflammation or infection, and no notable changes were observed in the fundus. Anterior segment optical coherence tomography (AS-OCT) (CASIA2 Advance, Tomey Corporation, Nagoya, Japan) confirmed PFM exposure on the conjunctiva.

**Video 1:** Aqueous leakage from the PFM confirmed by fluorescein staining. PFM: PreserFlo MicroShunt

**Figure 3:**
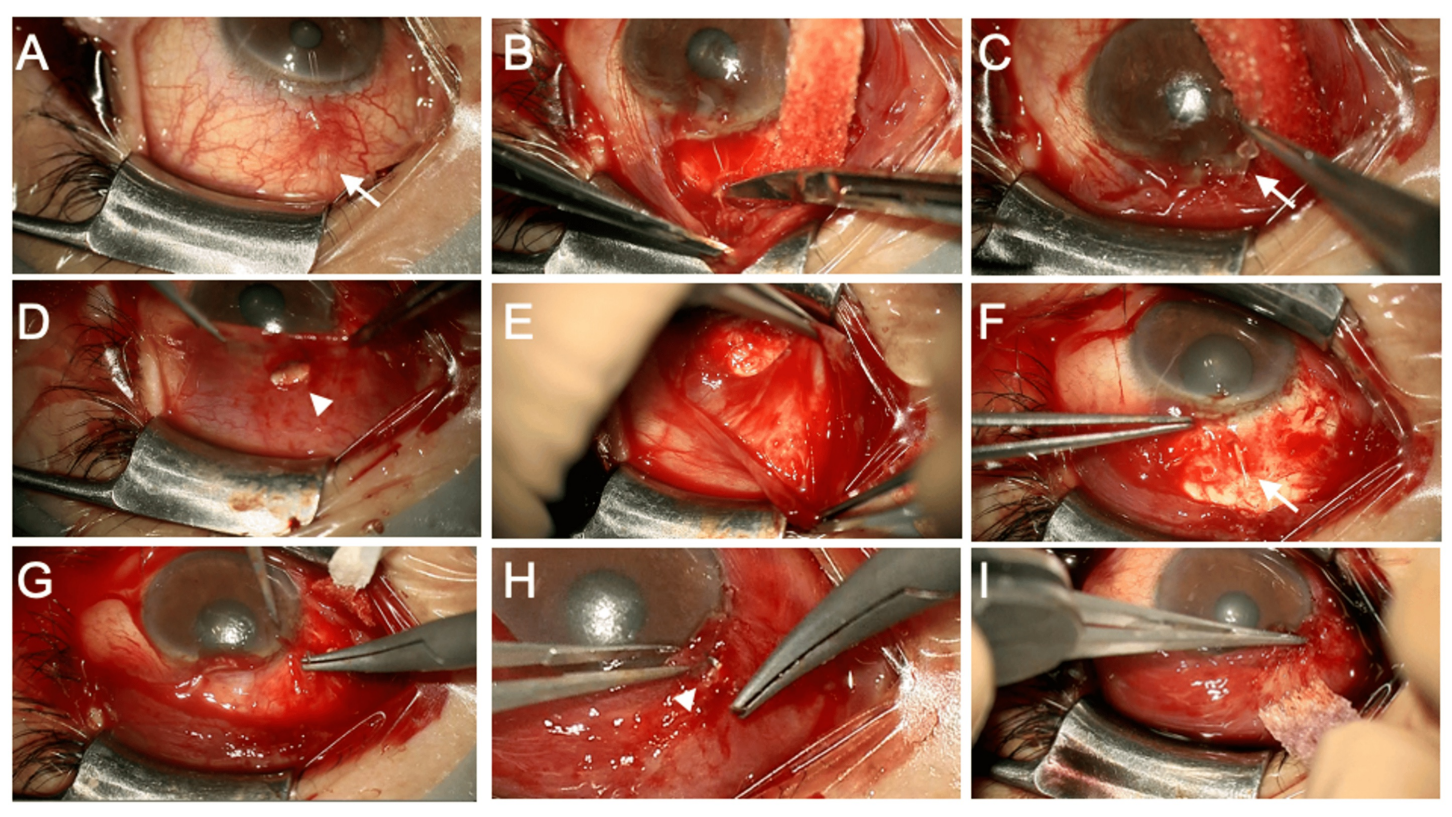
Surgical finding of conjunctival advancement procedure. (A) At the start of the surgery, the exposure of the PFM on the conjunctiva is observed (arrow). (B) Fornix-based conjunctival incision. (C) After dissecting the conjunctival adhesions surrounding the PFM, the device is temporarily removed (arrow). (D) The location of the conjunctival defect (arrowhead) is confirmed. (E) Tenon’s capsule is dissected in a membranous form from the conjunctiva. (F) After sufficiently separating the extensive adhesions between the conjunctiva, Tenon’s capsule, and sclera, the removed PFM is reinserted into the anterior chamber through the original scleral tunnel. (G) Tenon’s capsule is sutured to the corneal limbus. (H) The conjunctiva where the defect is located is advanced and sutured to the corneal limbus. (I) The conjunctival limbus is sutured, and the surgery is completed. PFM: PreserFlo MicroShunt

On the same day, conjunctival advancement surgery was performed to repair the exposed device (Figure 3A-3I and Video 2). Anesthesia was administered via sub-Tenon’s injection of 2% xylocaine. A conjunctival incision was made at the corneal limbus (Figure 3B), and the surrounding scar tissue was dissected. The PFM was temporarily removed (Figure 3C), and the conjunctival defect was measured (Figure 3D, arrowhead). The sclera was exposed over an area larger than one quadrant to allow for the advancement of the conjunctiva. Tenon’s capsule was dissected from the conjunctiva as a membranous form (Figure 3E). The PFM was reinserted into the scleral tunnel, and aqueous leakage was confirmed (Figure 3F, arrow). Tenon’s capsule was sutured to the corneal limbus with 10-0 Vicryl, followed by conjunctival suturing. To close the conjunctival defect, a suture was passed through the conjunctiva at the defect site and secured to the corneal limbus (Figure 3H, arrowhead). After subconjunctival dexamethasone injection and the confirmation of no conjunctival leakage, the surgery was completed (Figure 3I). Postoperatively, levofloxacin eye drops were administered four times a day for three weeks and betamethasone eye drops for six weeks.

**Video 2:** Surgical repair of PFM exposure using a conjunctival advancement procedure. PFM: PreserFlo MicroShunt

**Figure 4:**
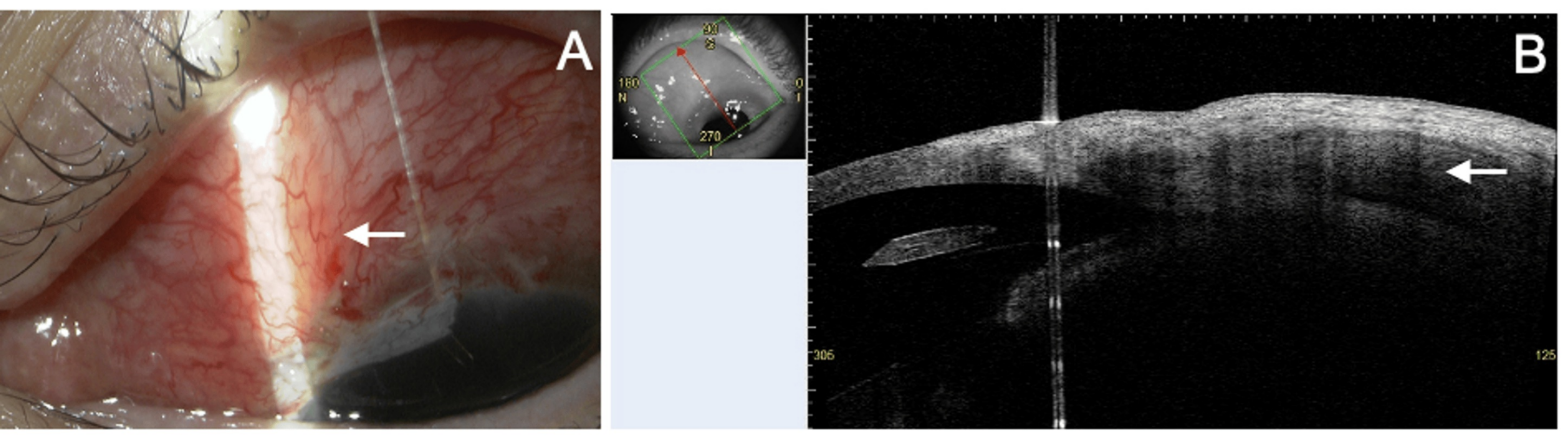
Slit lamp (A) and AS-OCT (B) findings two weeks after the repair surgery. (A) The PFM is covered by the conjunctiva, and the formation of a bleb is observed (arrow). (B) The formation of a bleb is confirmed by AS-OCT (arrow). The small image in the top left of this panel shows the scan direction. PFM, PreserFlo MicroShunt; AS-OCT, anterior segment optical coherence tomography

**Figure 5:**
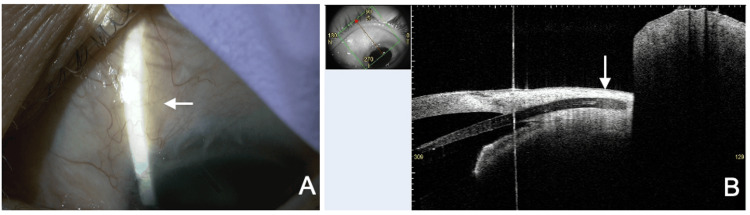
Slit lamp (A) and AS-OCT (B) findings 18 months after the repair surgery. (A) There is no recurrence of PFM exposure, but the bleb height has decreased compared to the early postoperative period (arrow). (B) AS-OCT shows that the device is covered by the conjunctiva (arrow). The small image in the top left of this panel shows the scan direction. PFM, PreserFlo MicroShunt; AS-OCT, anterior segment optical coherence tomography

Two weeks postoperatively, the BCVA in the left eye was 1.2, the IOP was 12 mmHg, and the anterior chamber was deep with no signs of inflammation. A well-formed bleb was observed (Figure 4A), and AS-OCT confirmed this (Figure 4B). One month after surgery, the corrected visual acuity in the left eye was 1.0, and the IOP was 22 mmHg. Latanoprost and timolol-dorzolamide eye drops were restarted. At the final follow-up, 18 months postoperatively, the corrected visual acuity in the left eye was 1.2, and the IOP was 13 mmHg. Although a decrease in bleb height was observed on slit lamp examination (Figure 5A) and AS-OCT (Figure 5B), no recurrence of device exposure was noted.

## Discussion

Previous reports of PFM device exposure have documented relatively early postoperative occurrences, ranging from one week to three months post-surgery [7,8]. In our case, device exposure was observed at six weeks postoperatively, consistent with these prior reports. Several risk factors for device exposure have been proposed, including prior glaucoma surgery [11], implantation in the inferior quadrant [11], the use of high-concentration MMC or the prolonged application of MMC [7], thin Tenon’s capsule [8], the loosening of nylon sutures [7], needling procedures [7], MMC-assisted bleb revision surgery [9], and severe blepharitis [8]. In our case, 0.04% MMC was applied for three minutes during the initial surgery, which is the standard protocol for Japanese patients. No needling procedures were performed, there were no signs of significant blepharitis or conjunctivitis, and the patient had no history of surgeries involving conjunctival incisions. Thus, no specific risk factors for exposure were identified in this case. However, early postoperative examination revealed mild conjunctival protrusion corresponding to the posterior end of the device, suggesting that the device may have been slightly elevated due to catching on Tenon’s capsule. It is possible that securing the device to the sclera with 10-0 nylon sutures during the initial surgery could have prevented exposure.

When device exposure occurs, surgical intervention is typically required, such as bleb revision, conjunctival suturing, or device removal. Simply suturing the conjunctiva may be insufficient due to the potential for recurrent exposure at the device edge, possibly caused by the contraction of the surrounding scar tissue. Therefore, when performing conjunctival suturing, it is important to sufficiently release and extensively dissect the scar tissue around the device. The scar tissue surrounding the PFM bleb typically shows the proliferation of fibroblasts and macrophages, but no significant signs of rejection have been reported [12]. This scar tissue is similar to that observed following trabeculectomy [12]. Thus, theoretically, bleb revision without device removal can reduce intraocular pressure (IOP) in a manner similar to that of trabeculectomy.

The technique described in this case aims to repair the device exposure while maintaining filtration efficacy by utilizing the same quadrant as the initial surgery. This approach has advantages over simple device removal or reimplantation in a different quadrant. Additionally, the absence of graft transplantation in this procedure makes it more generalizable. Regarding the outcomes of bleb revision after PFM implantation, one study reported that, in an analysis of 27 eyes, the IOP decreased from 26.4 mmHg preoperatively to 15.9 mmHg at one year postoperatively [13]. In our case, after 18 months, the bleb height remained low but stable, with a moderate reduction in IOP.

Successful conjunctival advancement requires sufficient adhesion release and adequate conjunctival mobility, with the added consideration that the conjunctival defect must not be too extensive. If, during surgery, it is determined that advancing the conjunctiva is not feasible, the procedure may need to be converted to simple device removal.

## Conclusions

Although exposure of the PFM device is a rare complication, it carries a risk of infection and requires prompt surgical intervention. In our case, conjunctival advancement surgery was performed to repair the exposed device, and during the 18-month follow-up period, no recurrence of exposure was observed, the bleb remained stable, and IOP was well-controlled. Conjunctival advancement surgery is an effective treatment option that allows for the repair of the exposure while preserving the filtration function of the device. When exposure occurs, it is crucial to sufficiently release the surrounding scar tissue and ensure complete coverage of the device.
